# The efficacy and safety of *Sipjeondaebo-tang* in Korean patients with cold hypersensitivity in the hands and feet: a protocol for a pilot, randomized, double-blind, placebo-controlled, parallel-group clinical trial

**DOI:** 10.1186/s13063-019-3286-7

**Published:** 2019-04-15

**Authors:** Youme Ko, Seung-Ho Sun, In-Sik Han, Ho-Yeon Go, Tae-Hun Kim, Jin-Moo Lee, Jun-Bok Jang, Kyoung Sun Park, Yun-Kyung Song, Kyou-Young Lee, Chan-Yong Jeon, Seong-Gyu Ko

**Affiliations:** 1 Department of Science in Korean Medicine, Graduate School, Kyung Hee University, 26 Kyungheedae-ro, Dongdaemun-gu, Seoul, Republic of Korea; 2 Department of Korean Internal Medicine, College of Korean Medicine, Sangji University, 80 Sangjidae-gil, Wonju-si, Gangwon-do 26339 Republic of Korea; 3 Department of Korean Internal Medicine, College of Korean Medicine, Semyung University, 65 Semyeong-ro, Jecheon-si, Chungcheongbuk-do Republic of Korea; 4 Korean Medicine Clinical Trial Center, College of Korean Medicine, Kyung Hee University, 23 Kyungheedae-ro, Dongdaemun-gu, Seoul, Republic of Korea; 5 Department of Korean Gynecology, College of Korean Medicine, Kyung Hee University, 26 Kyungheedae-ro, Dongdaemun-gu, Seoul, Republic of Korea; 6 Department of Korean Rehabilitation Medicine, College of Korean Medicine, Gachon University, 1342 Seongnamdae-ro, Sujeong-gu, Gyeonggi-do Republic of Korea; 7 Department of Ophthalmology, Otolaryngology, Dermatology, College of Korean Medicine, Sangji University, 80 Sangjidae-gil, Wonju-si, Gangwon-do 26339 Republic of Korea; 8 Department of Korean Internal Medicine, College of Korean Medicine, Gachon University, 1342 Seongnamdae-ro, Sujeong-gu, Gyeonggi-do 13120 Republic of Korea

**Keywords:** Herbal medicine, Cold hypersensitivity, *Sipjeondaebo-tang*, Cold intolerance

## Abstract

**Background:**

Cold hypersensitivity in the hands and feet (CHHF) is frequent in Asian countries including Korea. The quality of life can be degraded by the symptoms of CHHF. In particular, gynecological disorders such as menstrual pain, infertility, leucorrhea, and irregular bleeding may be related to CHHF. *Sipjeondaebo-tang* (SDT) is widely used in the treatment of various diseases including CHHF by balancing Yin and Yang, restoring the deterioration of physiological function, and improving immunity. However, the efficacy of SDT in the treatment of CHHF has not been assessed in clinical trials. Therefore, we aimed to investigate the feasibility of a full randomized clinical trial of SDT for the treatment of CHHF in Korean women through this trial.

**Methods:**

This study will be a pilot, randomized, double-blind, two-arm, placebo-controlled, parallel-group, multicenter clinical trial. Women aged 19–59 years who present with CHHF will be recruited from five university hospitals. A total of 60 subjects will be randomly assigned to a treatment group (SDT) or a placebo group at a 1:1 ratio. The subjects will receive 3 g of either SDT or placebo three times daily for 8 weeks. The primary outcome measures will be the Visual Analogue Scale scores of CHHF. The secondary outcome measures will be changes in body temperature in both the hands and the feet as measured using a thermometer and the Korean version of the World Health Organization Quality of Life Scale Abbreviated Version.

**Discussion:**

This will be the first trial to investigate the efficacy and safety of SDT in the treatment of CHHF. This study will provide basic clinical information regarding Korean herbal medicine treatment of CHHF and a clinical basis for designing a full randomized clinical trial.

**Trial registration:**

ClinicalTrials.gov, NCT03374345. Registered on 15 February 2018.

**Electronic supplementary material:**

The online version of this article (10.1186/s13063-019-3286-7) contains supplementary material, which is available to authorized users.

## Background

Cold hypersensitivity in the hands and feet (CHHF) is defined as a condition in which patients experience the sense of coldness in the hands and feet to a greater extent than do unaffected people in any environment, with worsening symptoms at lower temperatures [1]. Cold hypersensitivity is frequent in Asians, particularly among females. The ratio of females to males is about 3:2. [2]. Severely affected persons may feel uncomfortable when working with their hands, and the quality of life can deteriorate due to the discomfort of wearing socks or gloves in hot weather [3].

Studies have investigated the relationship between CHHF and diseases such as orthostatic hypotension [4], functional dyspepsia [5], and dysmenorrhea [6]. In particular, not only menstrual pain but also other gynecological disorders such as infertility, leucorrhea, and irregular bleeding may be related to CHHF according to Korean medicine [2]. The cause of CHHF might be related to various neurovascular, medical, psychosocial, environmental, and cultural factors [7]. However, the clear etiology of and appropriate treatment strategy for CHHF have not been identified, and current therapies are often associated with behavior modification [8]. Therefore, the establishment of effective treatment methods for CHHF is essential.

*Sipjeondaebo-tang* (SDT) is a frequently prescribed herbal formula in Korea, Japan, and China [9]. It is also called *Shi-Quan-Da-Bu-Tang* in China, and *Juzen-taiho-to* in Japan. SDT is used to treat both qi and blood deficiency syndrome by balancing Yin and Yang. It is also widely used in the treatment of chronic illnesses by restoring the deterioration of physiological function and improving immunity [10]. For example, SDT is clinically prescribed to treat anorexia, coldness of the hands and feet, weakness after illness, anemia, and night sweats [11]. Previous studies have suggested that SDT possesses a variety of biological properties including anti-cancer, anti-inflammatory, gastric protective action, and immune cell activation [12–15].

Although SDT is widely used in the treatment of various diseases including CHHF, no randomized clinical trial (RCT) has yet been performed to assess the clinical efficacy of SDT in the treatment of CHHF. Therefore, this randomized, placebo-controlled, double-blind trial aims to examine the feasibility of a full RCT of SDT for the treatment of CHHF in Korean female patients, and to evaluate the efficacy and safety of SDT.

## Methods

### Objectives

The general aim of this study is to evaluate the efficacy and safety of SDT in patients with CHHF.

The primary objective is to evaluate the efficacy of SDT by comparing the changes in VAS scores between the SDT and placebo groups after 8 weeks of administration.

The secondary objective is to assess the efficacy of SDT by comparing changes in body temperature (BT; thermometer measurement) and the Korean version of the World Health Organization Quality of Life Scale Abbreviated Version (WHOQOL-BREF) scores between the SDT and placebo groups.

### Study design and setting

This is a 12-week pilot, randomized, double-blind, two-arm, placebo-controlled, parallel-group, multicenter clinical trial that will be conducted in Gachon University Gil Korean Medical Hospital, Korean Medical Hospital of Sangji University, Semyung University Second Affiliated Korean Medical Hospital at Chungju, Kyung Hee University Korean Medical Center, and Kyung Hee University Korean Medicine Hospital at Gangdong in Korea.

**Fig. 1:**
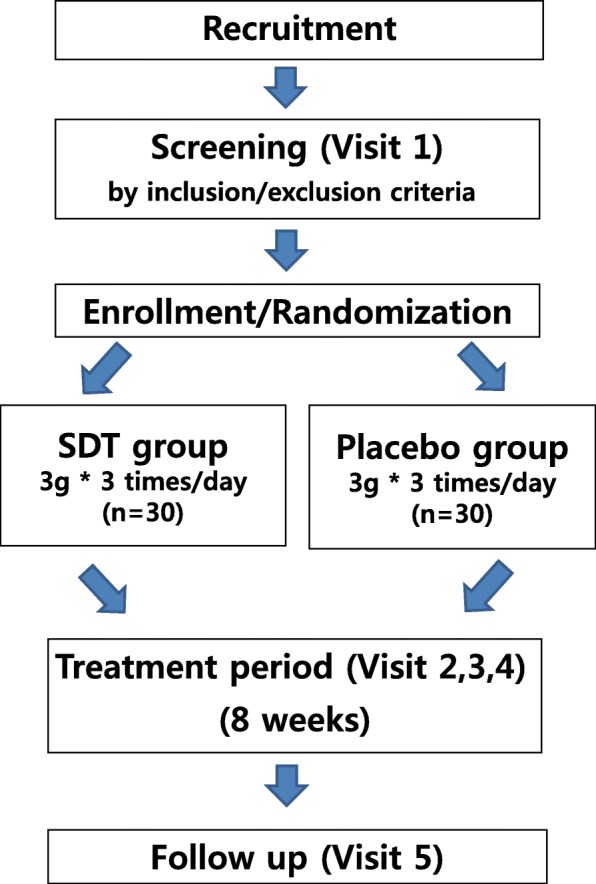
Participant timeline for the *Sipjeondaebo-tang* (SDT) study

A written informed consent will be obtained from each subject by the principal investigator (PI) or researcher after the subject has received sufficient explanation and a period of time in which to make a thoughtful decision. Each subject will be screened up to 7 days prior to randomization. Once they receive the trial subject identifier, they will be treated for 8 weeks and asked to visit the site every 4 weeks for safety and efficacy assessments. A follow-up visit will occur at 4 weeks after the end of the administration. The participants will be asked to return any unused investigational drugs at visit 3 and visit 4 in order to calculate drug compliance. During the trial, the participants will be prohibited from receiving other medications related to CHHF. The protocol design is based on the Consolidated Standards of Reporting Trials (CONSORT) guidelines and Standard Protocol Items: Recommendations for Interventional Trials (SPIRIT) checklist (see Additional file 1). Figure 1 presents a schematic flow diagram of the study.

### Participants

#### Inclusion criteria

The inclusion criteria are presented in Table 1. Participants must meet all of the following criteria.

**Table 1:** Inclusion criteria of the *Sipjeondaebo-tang* study

Inclusion criterion	
1. Women aged 19–59 years who present with CHHF	
2. Individuals who meet the following definition and have at least one of the following symptoms:	
2.1. Definition: those who feel cold in the hands or feet and experience discomfort	
2.2.1. Symptoms of CHHF at a temperature at which most individuals do not feel cold	
2.2.2. Symptoms of excessive CHHF at a temperature that one can feel cold	
2.2.3. Those who have symptoms of CHHF that do not recover easily when moving from a cold to a warm environment	
3. Those with a Visual Analogue Scale score of ≥ 4 cm at the screening visit	
4. At the screening visit, those who have a thermal difference of ≥ 0.3 °C between the palm (PC8) and the upper arm (LU4) as measured by a thermometer after room temperature adjustment at 24 ± 2 °C for 10 min.	
5. Those who can be observed during the clinical trial	
6. Those who agree to participate and provide written informed consent	

*CHHF* cold hypersensitivity in the hands and feet

### Exclusion criteria

The exclusion criteria of the SDT trial are presented in Table 2. Applicants who meet any of these conditions cannot participate in this study.

**Table 2:** Exclusion criteria of the trial

Exclusion criterion	
1. Patients receiving beta-blockers or calcium antagonists for the purpose of treatment of CHHF	
2. Patients with one or more ulcers or gangrene of the finger	
3. Those who have been diagnosed with hypothyroidism or prescribed thyroid medications	
4. Those who have been diagnosed with autoimmune diseases	
5. Those who have been diagnosed with carpal tunnel syndrome or have positive results on Phalen’s and Tinel’s tests	
6. Those who have been diagnosed with cervical disc herniation	
7. Those who have been diagnosed with diabetes mellitus	
8. Those receiving medicines that can affect the symptoms of CHHF, such as anticoagulants	
9. Those who present with CHHF due to postpartum or menopausal syndrome	
10. Those who present with a moderate level of liver dysfunction (AST and ALT levels of > 100 IU/L) or kidney dysfunction (creatinine level of > 2.0 mg/dl)	
11. Those who have behavior disorder, depression, anxiety, neurosis, or any other severe mental disorder	
12. Adult nonpregnant women with a hemoglobin level of < 7 g/dl and WBC count of > 11 cells × 10^9^ L	
13. Those whose mean SBP is ≥ 180 mmHg or DBP is ≥ 100 mmHg when measured twice	
14. Patients with an arrhythmia as determined by an electrocardiogram and requiring treatment, or with heart disease such as ischemic heart disease	
15. Alcohol or drug abusers	
16. Women who are pregnant (urine hCG positive), lactating women, and fertile women who have a pregnancy plan or do not consent to the proper method of contraception	
17. Those who have been diagnosed with a malignant tumor	
18. Those who are participating in other clinical trials	
19. Those who refuse to participate in clinical trials or provide written informed consent	
20. Those who cannot understand or speak Korean	
21. Those who are judged to be inappropriate for clinical trials by the researchers	

*ALT* alanine aminotransferase, *AST* aspartate aminotransferase, *CHHF* cold hypersensitivity in the hands and feet, *DBP* diastolic blood pressure, *hCG* human chorionic gonadotropin, *SBP* systolic blood pressure, *WBC* white blood cell

### Participant withdrawal criteria

Participants who meet the criteria summarized in Table 3 will be discontinued from the trial. The subjects who are withdrawn after randomization will be followed up for outcomes. Reasons for withdrawal will be documented in case report forms (CRF) and data will be analyzed using the intention-to-treat (ITT) principle. Each site research coordinator will be responsible for making next-visit reminder phone calls to prevent a participant’s withdrawal.

**Table 3:** Participant withdrawal criteria of the *Sipjeondaebo-tang* study

Participant withdrawal criterion	
1. Those whose medication compliance is < 70%	
2. Those who become pregnant during the study period	
3. Those who need surgery or inpatient treatment due to emergencies such as accidents or other illnesses	
4. Subject’s withdrawal of consent	
5. Subjects who have received prohibited medicines or therapies (e.g., anticoagulants, psychotropic drugs, and other drugs that may affect the symptoms of CHHF)	
6. Those who need standard treatment due to deterioration of symptoms of CHHF	
7. Occurrence of a serious adverse event	
8. Occurrence of factors making it difficult to sustain the process or investigator’s decision to terminate because of clinical trial results affected by some factors	

*CHHF* cold hypersensitivity in the hands and feet

### Randomization

Suitable participants, who agree to participate in this RCT, will be randomly assigned to a treatment group (SDT) or a placebo group at a 1:1 ratio. Thirty subjects will each be allocated to the SDT and placebo groups. A web-based randomization system (WBRS) developed by the contract research organization (CRO), the Institute of Safety and Effectiveness Evaluation for Korean Medicine (ISEE), will perform the assignment. The random order will be generated by the CRO’s independent professional statistician using the SAS 6.1 program and stratified by the hospital using random block sizes of two and four based on the allocation code provided by the SAS system.

### Blinding

Both the researchers and the subjects will be blinded to the assignment of study drugs. The sponsor’s CRO will label the randomization code number on the clinical trial drugs. The labeled trial products will be provided to clinical trial sites by the CRO. The same number of clinical trial medicines as the randomization number assigned via the WBRS will be administered to each subject by independent research pharmacists or assistants who are also blinded to the randomization, in each hospital. In the event of urgent medical conditions, such as serious adverse events (SAEs) or voluntary withdrawals, the principal investigator of the hospital will inform the CRO and sponsors immediately to process the unblinding in accordance with the ISEE’s standard operating procedures (SOPs).

### Procedure

#### Recruitment

Participants will be recruited from five Korean medicine university hospitals located in various cities in Korea. Each institution will recruit the persons who have visited each clinical institute for clinical trials or through public outdoor advertisement.

#### Study schedule

The items to be measured at each visit are presented in Table 4.

**Table 4:** Study schedule for the trial

	Screening	Treatment period			Follow-up
	Visit 1 (day − 7 to − 2)	Visit 2 (day 0)	Visit 3 (day 28 ± 3)	Visit 4 (day 56 ± 3)	Visit 5 (day 84 ± 3)
Informed consent	●				
Inclusion/exclusion criteria	●				
Randomization		●			
Drug compliance			●	●	
Vital signs	●	●	●	●	●
Body measurement^a^	●	●	●	●	●
Demographic, sociological, and gynecological information^b^	●				
Medical history^c^	●	●	●	●	●
General physical examination	●	●	●	●	●
Thermometer measurement^d^	●	●	●	●	●
Visual Analogue Scale	●	●	●	●	●
Adverse event monitoring			●	●	●
Pattern Identification Questionnaire	●				
WHOQOL-BREF		●		●	●
Laboratory tests^e^	●			●	
Chest X-ray scan and ECG	●				
Medication		●	●		
Blindness test				●	

*ECG* electrocardiogram, *WHOQOL-BREF* World Health Organization Quality of Life Scale Abbreviated Version

^a^Height and weight, but only weight for visit 2 and follow-up

^b^Age, job, digestion, exercise, smoking habit, drinking habit, sleep pattern, etc.

^c^Including general medical history and history related to cold hypersensitivity in the hands and feet

^d^Temperature measurement of both PC8 and LU4 at every visit and both ST32 and LR3 at visit 2

^e^Screening: hematological examination (white blood cells, red blood cells, hemoglobin, platelet), blood chemistry test (blood urea nitrogen, creatinine, aspartate aminotransferase, alanine aminotransferase, gamma-glutamyl transpeptidase, glucose), thyroid function test (free thyroxine, thyroid-stimulating hormone), urine test, pregnancy test (urine human chorionic gonadotropin). Visit 4: hematological examination (white blood cells, red blood cells, hemoglobin, platelet), blood chemistry test (blood urea nitrogen, creatinine, aspartate aminotransferase, alanine aminotransferase, gamma-glutamyl transpeptidase)

### Interventions

After randomization, the participants will be prescribed 3 g of SDT or placebo drug three times a day for a total of 8 weeks, with each dose taken before or between meals with warm water.

SDT and the placebo drug are produced by Hanpoong Pharm & Foods Co., Ltd. SDT granulated extract contains 1.0 g of Poria Sclerotium, 1.0 g of Cnidii Rhizoma, 1.0 g of Cinnamomi Ramulus, 1.0 g of Rehmanniae Radix Preparata, 1.0 g of Astragali Radix, 1.0 g of Paeoniae Radix, 1.0 g of Atractylodis Rhizoma Alba, 1.0 g of Ginseng Radix Alba, 1.0 g of Angelicae Gigantis Radix, and 0.5 g of Glycyrrhizae Radix. It will be extracted from the raw materials described and concentrated to 3 g per dose. The placebo granulated extract contains 1.7 g of lactose, 1.0 g of corn starch, 0.1 g of citric acid, 0.1 g of caramel coloring, and 0.1 g of *Ssanghwa* herbal flavor per dose. It has a similar color, shape, weight, flavor, and taste to those of SDT.

### Outcome

#### Primary outcome measure

The change in the VAS score between visit 2 (baseline) and visit 4 (after treatment) will be used as the primary outcome measure. The mean difference between the change scores of the two groups will be calculated.

#### Visual Analogue Scale

The VAS score (ranging from 0 cm as no coldness to 10 cm as the maximum coldness imaginable) will be used to assess the severity of CHHF. The VAS score will be measured at every visit.

#### Secondary outcome measure

The change in the BT and WHOQOL-BREF score between visit 2 (baseline) and visit 4 (after treatment) will be used as the secondary outcome measure. The mean difference between the change scores of the two groups will be calculated.

#### BT: thermometer measurement

After 20 min of relaxation at 24 ± 2 °C, the BT will be measured by thermometer (835-T1; Testo, Lenzkirch, Germany) at acupoints PC8 and LU4 at every visit and at ST32 and LR3 at visits 2, 3, 4, and 5.

#### WHOQOL-BREF

The Korean version of the WHOQOL-BREF is comprised of a 26-item questionnaire estimating quality of life. It assesses five areas: general quality of life, psychological health, physical health, environmental health, and social relationships [16]. The WHOQOL-BREF will be conducted at visits 2, 4, and 5.

#### Safety outcome measure

The safety assessment will be performed for all subjects who have been randomized and received study medication more than once. The subjects’ vital signs and general physical status will be examined at every visit. Hematological examination (WBC, RBC, hemoglobin, platelet) and blood chemistry tests (blood urea nitrogen, creatinine, AST, ALT, gamma-glutamyl transpeptidase (γ-GTP)) will be performed at visits 1 and 4. Blood sugar test, thyroid function test (free T4, TSH), urine test, and pregnancy test (urine human chorionic gonadotropin) will be conducted at visit 1. The occurrence of adverse events (AEs) will be checked at visits 3, 4, and 5.

#### Compliance calculation

A total of 93 investigational drugs will be provided to the subjects at visit 2 and visit 3. The subjects will be asked to return the remaining investigational medications at visit 3 and visit 4 for the purpose of calculating drug compliance. The rate of compliance will be calculated as: compliance (%) = [93 – remaining products / expected intake] × 100. Clinical trials will be continued only if compliance is ≥ 70.

#### AE reporting

The investigators should educate the subjects to report any AEs that occur after administration. All adverse events that occur after the start of this trial should be recorded in the case report form whether or not they are related to the test agent. All AEs will be evaluated for causal relationships. When SAEs occur, the researchers are required to notify the Institutional Review Board (IRB) and regulatory authorities within 24 h.

#### Sample size calculation

The change in the VAS score between visit 2 (baseline) and visit 4 (after treatment) will be used as the primary outcome measure. The hypotheses are as follows:

- H0: δ = Δ1 – Δ2 = 0
- H1: δ = Δ1 – Δ2 ≠ 0

where Δ1 is the average change in the VAS score between visit 4 and visit 2 in the SDT group, and Δ2 is the average change in the VAS score between visit 4 and visit 2 in the placebo group

Based on a similar study [17], the expected average VAS score difference in the SDT group is 11.85 (larger than 10, because in the study used as a reference a 100-mm VAS was applied to assess severity of CHHF, and the scores ranged from 0 to 100), and the standard deviation (SD) is estimated to be 15.255 using the pooled standard deviation formula. Considering a dropout rate of 13%, which is agreed among medical experts, we need to recruit 30 subjects per arm to calculate the treatment efficacy, and to offer the power calculation for a future large-scale RCT.

### Statistical analysis

#### Efficacy assessment

For the efficacy assessment of this trial, both intention-to-treat (ITT) and per-protocol (PP) analyses will be performed. Missing data will be adjusted using the last-observation-carried-forward (LOCF) imputation method on ITT analysis. For the safety assessment of this trial, PP analysis will be conducted. The continuous variables will be expressed as the mean ± SD and the nominal variables will be reported as percentages. The ITT method will include all randomly assigned subjects, regardless of any protocol violations or study dropouts. The PP method will include only the subjects who have completed the 12-week study period without any major protocol violations and who have a compliance rate of ≥ 70%. The baseline characteristics will be compared by either a chi-square test for nominal variables or analysis of variance (ANOVA) for continuous variables. For the within-group analysis, the primary and secondary outcome variables will be assessed using a paired *t* test. The between-groups analysis after 8 weeks of administration will be performed using a repeated-measure ANOVA for the primary outcome variables, and a paired *t* test for the mean difference in the VAS score between the SDT group and the placebo group. As a secondary outcome variable, the mean differences in BT and WHOQOL-BREF scores between the groups will be analyzed using a repeated-measure ANOVA for BT and an ANOVA and post-hoc analysis for the WHOQOL-BREF scores. For all non-normal distribution data, a nonparametric statistical test will be performed. SPSS for windows version 23.0 (SPSS Inc., Chicago, IL, USA) will be used for statistical analysis. The statistical significance level will be set at *P* < 0.05.

#### Safety assessment

Safety analysis will be conducted on all subjects who will be randomized and visited at least once after screening. Safety-related measures will be analyzed using the ITT method. The safety data will be stratified according to the symptoms.

### Data management and monitoring

All records will be collected in paper case report form (CRF) files. To protect confidentiality, the files are stored in a secure and locked place and manner. The subject identification and privacy information will be deleted from all study documents. Once the trial is completed, a double independent data entry will be performed for promoting the data quality. After finishing the data entry and dealing with the query, the database will be locked and analyzed by an independent statistician of the ISEE under the confirmation of the PI. Site investigators will have direct access to the final data sets from their own sites.

Monitoring will be performed by the CRO (the ISEE). Monitoring will begin after the first participant completes the entire course of the study. All institutions conducting clinical trials will be monitored in accordance with the SOPs during the course of the clinical trials. Range checks for data values and double data entry will be performed to improve the data quality. No auditing will be performed for this trial.

## Discussion

CHHF has close relationships with Raynaud’s phenomenon (RP) as they both have common symptoms of coldness in the hands and feet. According to one study, 43% of CHHF patients in Korea had RP [18], and many family practitioners prescribe medicines for CHHF according to the treatment recommendations for RP [19]. These reports indicate that CHHF can be considered a potential symptom of RP.

RP refers to transient vasospasm of the peripheral arteries, with pallor changes followed by cyanosis and erythema. It is reported that 3–5% of the population can be affected, and that RP can be hereditary [20]. RP is categorized as primary or secondary RP, depending on the presence or absence of underlying diseases such as connective tissue disease [21]. The pathophysiology of RP is not yet clearly understood. A major factor contributing to the development of RP is considered to be the dysregulation of vasomotor activity, which results in an imbalance between vasoconstriction and vasodilation [22].

Traditional herbal medicine is commonly used in the treatment of CHHF in Korea. According to traditional Korean medicine, CHHF is one symptom constituting the cold syndrome pattern. Therefore, Korean medicine is not only aimed at symptom improvement, but is also prescribed to balance the whole body [23].

SDT is a frequently prescribed herbal formula in Korea. Chen Shiwen first described it in *Prescriptions from the Great Peace Imperial Grace Pharmacy*, the classic Chinese medical book from 1107 [24]. SDT consists of 10 herbs: Poria Sclerotium, Cnidii Rhizoma, Cinnamomi Ramulus, Rehmanniae Radix Preparata, Astragali Radix, Paeoniae Radix, Atractylodis Rhizoma Alba, Ginseng Radix Alba, Angelicae Gigantis Radix, and Glycyrrhizae Radix [25]. It is commonly used for the treatment of CHHF by tonifying the qi and blood [24]. In addition, the classic Chinese medical text *The Yellow Emperor’s Inner Classic* describes the notion of the “spleen and four extremities,” which explains that the spleen functions to regulate the four limbs [26, 27]. Through this notion, limb dysfunction is therefore indicative of splenic problems, and we can postulate that frequent use of SDT for the treatment of CHHF is related to the regulation of spleen function with gastric protective action. Despite the wide use of SDT in the treatment of various diseases including CHHF, no RCT has been performed to assess the clinical efficacy of SDT in the treatment of CHHF. Therefore, evaluation of the efficacy and safety of SDT in the treatment of CHHF is necessary.

For evaluation of the outcome, the VAS score will be used as a primary outcome measure, and the BT as measured by thermometer and the WHOQOL-BREF, a questionnaire estimating quality of life, will be used as secondary outcome measures. There are some limitations of this study. The first is the limited use of outcome measurement methods. Outcome measures such as infrared thermography, the cold stress test (CST), distal–dorsal difference assessment, and heart rate variability analysis have not been applied. Another limitation is the absence of laboratory tests to exclude RP. Due to difficulties in acquiring accurate measurements, RP is likely to be misdiagnosed as a symptom of CHHF. Our team previously performed a pilot study [28] of CHHF and *Danggui-SayukGa-Osuyu-Saenggang-tang*, and conducted the CST and antinuclear antibody test to exclude RP patients. However, very few subjects were excluded by the test, and only the expenditure of the research grant was increased. Therefore, in the current trial, we will not include these tests. In the near future, our team will prepare for further large-scale clinical trials searching for methods to accurately distinguish CHHF from primary RP. Despite these limitations, as the first study to assess the efficacy and safety of SDT in the treatment of CHHF, we expect that this study will provide basic clinical information regarding Korean herbal medicine treatment of CHHF and that this study protocol will be the basis for designing a full RCT.

## Trial status

Participant recruitment began on 31 January 2018, and 50 participants have been recruited until now. Recruitment will complete in May 2020. The protocol version 1.2 (1 December 2018) is currently active.

## Additional files

Additional file 1: SPIRIT 2013 checklist: recommended items to address in a clinical trial protocol and related documents (PDF 39 kb)

## Abbreviations

AE: Adverse event
ALT: Alanine aminotransferase
ANOVA: Analysis of variance
AST: Aspartate aminotransferase
BT: Body temperature
CHHF: Cold hypersensitivity in the hands and feet
CRO: Contract research organization
CST: Cold stress test
DBP: Diastolic blood pressure
hCG: Human chorionic gonadotropin
ISEE: Institute of Safety and Effectiveness Evaluation
ITT: Intention to treat
PP: Per protocol
RBC: Red blood cell
RCT: Randomized clinical trial
RP: Raynaud’s phenomenon
SAE: Serious adverse event
SBP: Systolic blood pressure
SD: Standard deviation
SDT: *Sipjeondaebo-tang*
SOP: Standard operating procedure
TSH: Thyroid-stimulating hormone
VAS: Visual Analogue Scale
WBC: White blood cell
WBRS: Web-based randomization system
WHOQOL-BREF: World Health Organization Quality of Life Scale Abbreviated Version
